# Transcranial Magnetic Stimulation as an Interventional Tool for Addiction

**DOI:** 10.3389/fnins.2020.592343

**Published:** 2020-10-22

**Authors:** Vaughn R. Steele

**Affiliations:** Department of Psychiatry, School of Medicine, Yale University, New Haven, CT, United States

**Keywords:** addiction, rTMS, iTBS, cTBS, known unknowns

Repetitive transcranial magnetic stimulation (rTMS) is implemented to treat many clinical diagnoses. The most common clinical patients receiving rTMS are those suffering from treatment resistant depression (TRD). As a treatment for TRD, rTMS is thought to modulate circuits dysregulated by the disease (Fox et al., 2012). Considering many clinical populations have overlapping dysregulated circuits (Goodkind et al., 2015; McTeague et al., 2017), rTMS holds tremendous potential to treat myriad diseases. One such disease that is manifest by dysregulated circuits is substance use disorder (SUD) (Volkow et al., 2016). The dysregulation spawns from the mesocorticolimbic dopamine (MCL-DA) system and linked to initiation and maintenance of addictive behaviors (Goldstein and Volkow, 2002). Drug use increases DA release in MCL-DA system (Jay, 2003; Kelley, 2004; Nestler, 2005), which is thought to be an important element in learning, goal-directed behavior, and reward processing (Everitt and Robbins, 2005; Kalivas and O'Brien, 2008). The MCL-DA system was modulated with repeated drug exposures to increase dysregulations in SUDs. Cortical rTMS modulates dopamine release in the MCL-DA (Strafella et al., 2001; Strafella, 2003) suggesting rTMS has therapeutic potential for clinical disorders related to DA, such as SUDs.

## Overview of the Special Issue

Recently, a large number of researchers are testing the potential of rTMS to treat SUDs (Diana et al., 2017) and the field is beginning to coalesce toward specific methodological approaches in this regard (Ekhtiari et al., 2019). As with any new field of study, there are many “known unknowns” to uncover to optimize treatment application and increase positive outcomes (i.e., reduce relapse). The topic of this special issue of *Frontiers in Neuroscience* is a timely and important one with a set of papers gathered together that are provocative and wide-ranging. They advance our knowledge by tackling a few known unknowns of how rTMS could be applied to address the negative impact of SUDs on society. The 11 papers range from empirical studies with rTMS applied to treat cocaine, methamphetamine, alcohol, and eating disorders; reviews on rTMS as a treatment for cocaine, methamphetamine, amphetamine, and gambling disorder; and two commentaries discussing the potential of motor cortex stimulation as a target site for intervention. Specifically, motor cortex excitability, and the relationship to glutamate (Nardone et al., 2019), could be used to assess and increase inhibitory control known to be dysregulated in SUDs (Volkow et al., 2016; Zilverstand et al., 2018), as a treatment for alcohol use disorder (AUD) and SUDs in general (Zhou et al., 2019).

The systematic reviews include rTMS studies applied to treat SUD and gambling disorder. Although there are few published studies using rTMS or transcranial direct current stimulation (tDCS), the research topic is ripe for investigation (Zucchella et al., 2020). Gambling disorder has similar behavioral and pathophysiological manifestations as SUDs, suggesting potential overlap in dysregulation and interventional tools for treatment. In treating SUDs with rTMS, these reviews highlight the importance of considering both dopamine and glutamate (Moretti et al., 2020) as well as assessing individual differences in patients (Ma et al., 2019) to better uncover the known unknown of the underlying mechanisms of rTMS interventions. Also, applying high frequency stimulation (~5–20 Hz) is more effective than low frequency (~1 Hz) at reducing craving post-rTMS (Ma et al., 2019). This reflects the shift toward implementing the high frequency, and shorter to implement, continuous and intermittent theta-burst stimulation (c/iTBS) protocols (Huang et al., 2005) than other rTMS protocols.

To implement rTMS in a SUD sample, many target dorsolateral prefrontal cortex (dlPFC) or the medial prefrontal cortex (mPC). Preclinical models of optogenetic stimulation helped motivate these stimulation sites (Chen et al., 2013). In humans, these sites could also be selected to intervene and treat impulsivity, inhibitory control, executive functioning deficits in addiction (c.f., Zilverstand et al., 2018). Additional preclinical models with recent focal coil developments (Meng et al., 2018; Cermak et al., 2020) will continue to influence human rTMS applications. Such models are extremely useful when targeting known aberrant pathways with rTMS. Moretti et al. (2020) outlined the glutamatergic pathway between PFC and nucleus accumbens as one such pathway in need of rigorous study as it is essential for compulsive drug-seeking behaviors.

A fundamental question when implementing rTMS as a treatment is whether cognitive functions are modified. Schluter et al. (2019) implemented an active/sham 10 Hz rTMS treatment protocol in AUD participants and measured cognitive functions before and after the intervention. Although this first randomized clinical trial using AUD and rTMS did not report significant change in the targeted and measured cognitive functions, the authors demonstrated feasibility of applying chronic rTMS to an AUD sample safely. Also, the authors measured a proximal and targeted cognitive function instead of a distal, and common measure of craving as a metric of treatment success.

Known unknowns when applying rTMS to treat SUDs also include which location, which hemisphere, and which stimulation protocol should be selected. Empirically, Sanna et al. (2019) tested whether bilateral 15 Hz rTMS and iTBS applied to the dlPFC differed in effectively reducing cocaine craving. Both treatments reduced craving similarly suggesting the faster iTBS would be easier and more cost effective to apply in a clinical setting. In a methamphetamine treatment seeking sample, Zhao et al. (2020) reported reduced craving in each group that received one of three rTMS dlPFC protocols (left iTBS, left cTBS, or right cTBS) suggesting any TBS intervention could be effective. This adds to recent findings that the general historical understanding that stimulation protocols have opposite effects (Pascual-Leone et al., 1998; Huang et al., 2005) suggesting effects are not universal (Liu et al., 2020; Steele, 2020). Steele et al. (2019) applied accelerated iTBS treatment to the left-dlPFC in cocaine users while they viewed cocaine cues to engage the targeted circuit. Participants reported reduced use (both amount and frequency) 1-month post-treatment. The importance of measuring and reporting off-target effects is demonstrated by the Zhao et al., and Steele et al. articles. Mood, sleep, and anxiety scores improved in the methamphetamine sample by Zhao et al., mood improved and reduced use of other substances were found in the cocaine sample by Steele et al. These measures should be collected and reported as off-target effects related to all rTMS treatments of clinical populations.

The final known unknown addressed in this special issue relates to the state of the participant while receiving the rTMS intervention. Could the state (e.g., physiological, cognitive) of the participant facilitate the effectiveness of the treatment? Stramba-Badiale et al. (2020) outlined how virtual reality (VR) could be implemented in conjunction with rTMS during treatment sessions for eating disorders. This is a very promising development and an exciting area for study with the potential of combining the two interventions to be more effective than each applied serially. Pharmacological interventions could also be considered (Spagnolo et al., 2020). Accounting for the state of the patient will likely prove to be an important variable when developing an effective treatment for SUDs.

## Conclusion

The articles included in this special issue brought us closer to developing an understanding of how to move forward in using rTMS as a therapeutic intervention for addiction. There are promising results and tantalizing effects to drive thorough research into uncovering more known unknowns. Generally, rTMS used to treat SUDs was tolerated by a wide range of patients. Applying chronic rTMS as a treatment also proved feasible and generally effective at modifying the targeted behavior. Higher frequency stimulation produced greater benefits to the patient. This is all good news and is in line with a recent consensus paper outlining steps toward developing an rTMS treatment for SUDs (Ekhtiari et al., 2019). Some of the most interesting known unknowns are likely soon to be uncovered. Specifically, researchers are diligently working to understand the mechanisms of change induced by rTMS and the effects related to inherent individual differences in patient populations. Also, there is growing evidence that an engaged circuit is beneficial toward positive outcomes (e.g., VR in Stramba-Badiale et al., 2020) and cocaine cues in Steele et al. (2019).

Future directions are apparent from this special issue. Foundational experiments are essential in three areas: (1) develop and integrate preclinical models to clinical applications of rTMS, (2) elucidate effective combinations of rTMS and other treatments, (3) identify individual differences with respect to inducing excitation and inhibition with rTMS. Recent coil technology allows focal stimulation in rodents (Meng et al., 2018) which should speed the parameter space search in optimizing rTMS applications. Also, developing realistic preclinical models could help uncover the true rTMS mechanism of action related to neuroplastic change thus optimizing clinical rTMS applications. Combining rTMS with other interventions (either behavioral or pharmacological) is a promising area of research that should be systematically explored as it could improve treatment outcomes beyond any single intervention (c.f., Spagnolo et al., 2020). Finally, it is essential to understand the universality, or non-universality, of “excitatory” and “inhibitory” rTMS sequences. These individual differences on how rTMS induces neuroplastic change is the largest factor in applying rTMS within-participant as an effective treatment for SUDs and other clinical population (Steele, 2020), Together, this special issue highlights future directions for the field to explore to evaluate whether rTMS is an effective treatment for SUDs. New findings are rapidly immerging in this exciting area of research. It is only a matter of time before the field uncovers enough known unknowns to implement an optimized therapeutic rTMS intervention for SUDs.

## Author Contributions

VRS conceived and wrote this commentary.

## Conflict of Interest

The author declares that the research was conducted in the absence of any commercial or financial relationships that could be construed as a potential conflict of interest.
